# Bridge under troubled water: Turbulence and niche partitioning in fish foraging

**DOI:** 10.1002/ece3.2593

**Published:** 2016-11-25

**Authors:** Zeynep Pekcan‐Hekim, Noora Hellén, Laura Härkönen, Per Anders Nilsson, Leena Nurminen, Jukka Horppila

**Affiliations:** ^1^ Department of Environmental SciencesUniversity of HelsinkiHelsinkiFinland; ^2^ Department of Biology ‐ Aquatic EcologyLund UniversityLundSweden; ^3^ Department of Environmental and Life Sciences ‐ BiologyKarlstad UniversityKarlstadSweden

**Keywords:** competition, diet choice, disturbance, feeding strategy, segregation

## Abstract

The coexistence of competing species relies on niche partitioning. Competitive exclusion is likely inevitable at high niche overlap, but such divide between competitors may be bridged if environmental circumstances displace competitor niches to enhance partitioning. Foraging‐niche dimension can be influenced by environmental characteristics, and if competitors react differently to such conditions, coexistence can be facilitated. We here experimentally approach the partitioning effects of environmental conditions by evaluating the influence of water turbulence on foraging‐niche responses in two competing fish species, Eurasian perch *Perca fluviatilis* and roach *Rutilus rutilus*, selecting from planktonic and benthic prey. In the absence of turbulence, both fish species showed high selectivity for benthic chironomid larvae. *R. rutilus* fed almost exclusively on zoobenthos, whereas *P. fluviatilis* complemented the benthic diet with zooplankton (mainly copepods). In turbulent water, on the other hand, the foraging‐niche widths of both *R. rutilus* and *P. fluviatilis* increased, while their diet overlap simultaneously decreased, caused by 20% of the *R. rutilus* individuals turning to planktonic (mainly bosminids) prey, and by *P. fluviatilis* increasing foraging on littoral/benthic food sources. We show that moderate physical disturbance of environments, such as turbulence, can enhance niche partitioning and thereby coexistence of competing foragers. Turbulence affects prey but not fish swimming capacities, with consequences for prey‐specific distributions and encounter rates with fish of different foraging strategies (pause‐travel *P. fluviatilis* and cruise *R. rutilus*). Water turbulence and prey community structure should hereby affect competitive interaction strengths among fish species, with consequences for coexistence probability as well as community and system compositions.

## 1 Introduction

Competition is a major feature characterizing inter‐ and intraspecific interactions in animal communities. Different species or groups within species can compete for limited resources such as food or space (Sih, Crowley, McPeek, Petranka, & Strohmeier, 1985; Tilman, 1982), and competition may lead to changes in fecundity, individual growth, age structure, or population density (Dunham, 1980; Jones & Barmuta, 1998; Petren & Case, 1996). If species exploit common resources, the species that can maintain a positive per capita growth rate at the lowest resource level can drive other species extinct (Amarasekare, 2003; Gause, 1934). The coexistence of species therefore requires niche difference or partitioning. Niche partitioning can occur by specializing on certain resources, different timing of resource exploitation, or habitat segregation (Chesson, 2000; Tilman, 1982). Thus, competition can lead to habitat shifts and/or diet changes (Svanbäck & Bolnick, 2007). Such effects of competition have been found in plants, mammals, lizards, and birds, as well as in fish populations (Conradt, Clutton‐Brock, & Thomson, 1999; Lack, 1971; Pianka & Huey, 1978; Svanbäck & Bolnick, 2007; Urban, Tewksbury, & Sheldon, 2012; Werner & Hall, 1977).

The interaction strength between coexisting competitor species can be affected by altered environmental factors if the species differ in their responses to disturbances. Disturbances are defined as changes in factors external to the level of interest, occurring at different spatial and temporal scales (Pickett, Kolasa, Arnesto, & Collins, 1989). In aquatic ecosystems, feeding efficiencies of predators, and thus also their competitive interactions, can be affected by visual or physical disturbances. One commonly studied disturbance is decreased water transparency due to eutrophication, increasing loads of suspended solids, or dissolved organic matter (Jeppesen, Søndergaard, & Jensen, 2003; Wrona et al., 2006), where changes in transparency affect competitive interaction strength according to competitor reliance on visual cues for foraging (Diehl, 1988; Estlander et al., 2010). Physical disturbance such as turbulence, the irregular, diffusive, dissipative flow of water, may also affect competition, but is largely understudied. Most flows occurring in nature are turbulent (Tennekes & Lumley, 1972), and may be caused by a variety of processes in aquatic systems, including wind stress, buoyancy flux, breaking internal waves, and drag at water–sediment boundary layers (Imboden & Wüest, 1995). Turbulence can, for instance, disperse planktonic animals and thereby affect encounter rates between planktivores and their prey (Baranyai, G.‐Toth, Vári, & Homonnay, 2011; Joensuu, Pekcan‐Hekim, Hellèn, & Horppila, 2013; MacKenzie, Miller, Cyr, & Leggett, 1994; Rothschild & Osborn, 1988). As prey selection by fish is often density‐dependent and species‐specific (e.g., Maszczyk & Gliwicz, 2014; Werner & Hall, 1974), turbulence may affect encounter rates, prey selectivity, and thereby competitive interactions between fish species. Moreover, turbulence could affect encounter rates differently between foraging strategies of competitors (e.g., cruising predators vs. pause‐travel predators; MacKenzie & Kiørboe, 1995), with consequences for interaction strengths between coexisting competitors.

The effects of turbulence depend on the relative swimming speeds of predators and prey, and are most pronounced when the difference is small (Rothschild & Osborn, 1988). Because larger‐sized predators have high maneuverability and swimming velocity, it has been assumed that turbulence unlikely affects their foraging rates (Kiørboe & Saiz, 1995). Therefore, in aquatic systems turbulence has been considered important only for invertebrate predators and small, larval fish. Additionally, previous turbulence/predation studies have focused on planktivory only (Kiørboe & Saiz, 1995; MacKenzie & Leggett, 1991; Pekcan‐Hekim, Joensuu, & Horppila, 2013; Rothschild & Osborn, 1988). Turbulence‐dependent competition between foragers in a more natural occurrence of both planktonic and benthic prey has hitherto received little attention. The effects of water flow on niche partitioning of fish in lotic waters have been studied (e.g., Lee & Suen, 2012), but effects of turbulence in lentic ecosystems have been much less frequently considered. Many fish species switch between planktonic and benthic feeding depending on relative availability (Lammens, de Nie, Vijverberg, & van Densen, 1985; Uusitalo et al., 2003). Turbulence could hereby affect the choice between benthic and planktonic prey by changing the propensity for zooplanktivory.

The level of turbulence in lake habitats is affected by both wind speed and water depth. The intensity of turbulence does depend not only on the energy input flux by wind, but also on the vertical space available for energy dissipation (G.‐Tóth, Parpala, Balogh, Tátrai, & Baranyai, 2011; Tennekes & Lumley, 1972). In shallow water bodies, turbulence has only little space to dissipate. Therefore, the turbulent energy content and turbulent shear forces in lakes can be higher than in the ocean (G.‐Tóth et al., 2011). Climate models predict increasing wind speeds in northern Europe, with consequences for turbulence in aquatic ecosystems (Samuelsson, 2010). The predicted reductions in water level of many lakes can moreover affect the turbulence conditions by reduced vertical space for energy dissipation (Ficke, Myrick, & Hansen, 2007; G.‐Tóth et al., 2011). Therefore, turbulence is likely to have a particularly strong regulatory role together with other physical–chemical factors such as temperature, light, and pH that are already well known to influence lake ecosystems.

**Figure 1:**
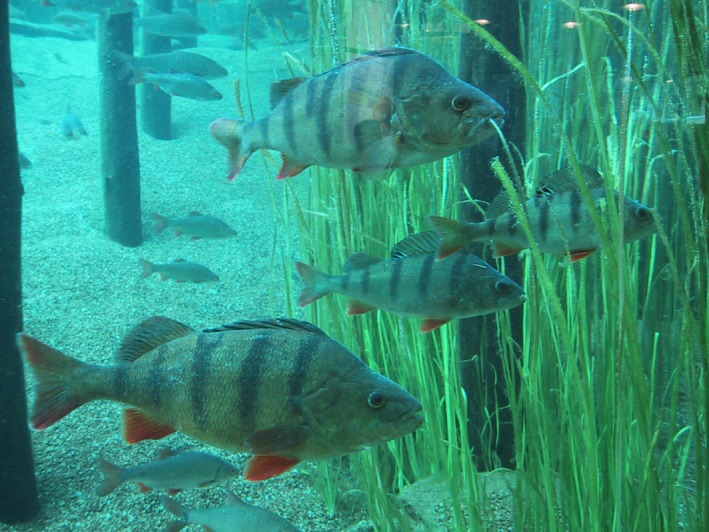
A shoal of perch (in front) and roach (in the back). © Leena Nurminen

We here experimentally investigate the effect of water turbulence on the niche partitioning of two common and influential predator fish species. Eurasian perch (*Perca fluviatilis* L.) and roach (*Rutilus rutilus* (L.) (Figure 1) are widely distributed in Europe, coexisting and dominating many types of natural lentic habitats (Mehner, Diekmann, Brämick, & Lemcke, 2005; Olin et al., 2002; Persson, 1986; Rask, Viljanen, & Sarvala, 1999). In numerous small lakes, they are the only planktivorous/benthivorous fish species (Rask et al., 2000). Both perch and roach feed on zooplankton at juvenile stages, but with increasing size perch switch to benthic macroinvertebrates and later to a piscivorous diet, while large roach feed on zooplankton, benthic macroinvertebrates, plant material, and detritus (Horppila et al., 2000; Kahl & Radke, 2006; Persson, 1983). Many studies have demonstrated that roach is a more efficient planktivore than perch, while perch is the superior benthic feeder (e.g., Bergman, 1990; Persson, 1983, 1987). It has been suggested that intensive consumption of zooplankton by roach increases intraspecific competition for zoobenthos in perch populations (Persson & Greenberg, 1990). It has however also been suggested that perch has a higher prey capture rate on copepod prey than roach and that diverging and inconclusive results originate from differences in experimental circumstances (Peterka & Matĕna, 2009, 2011). Effects of water turbulence on foraging competition and food selectivity were neglected in these previous studies.

Turbulence likely has different effects on foraging in perch and roach, as they use different feeding strategies; perch uses a pause‐traveling feeding mode, while roach is a cruise predator (e.g., Peterka & Matĕna, 2011). Pause‐travel predators rely primarily on prey motility and are discontinuous swimmers, whereas cruise predators continually move to locate prey (Greene, 1986). As turbulence can increase plankton movement, pause‐travel predators may benefit from turbulence as they are stationary during the encounter process, whereas for cruise predators the effect of turbulence may not be prominent (MacKenzie & Kiørboe, 1995). We therefore hypothesized that turbulence has a more positive effect on zooplanktivorous feeding of perch compared with roach (Estlander et al., 2010). Consequently, we also hypothesized that turbulence enhances niche partitioning between perch and roach by reducing competitive interaction strength.

## 2 Materials and Methods

### 2.1 Experimental setup

The study was conducted in June 2012 in experimental outdoor ponds (each 8.1 m^2^ surface area, rectangular shape, 3,200 L volume, max depth 60 cm, average depth 40 cm) at the Evo Field station of the Finnish Game and Fisheries Research Institute in southern Finland (61°13′N 25°12′E). The ponds had sand–gravel bottom with a 0.5‐ to 1‐cm layer of organic debris. The ponds mimic circumstances in natural lakes, and the shallow depth prevents zooplankton from escaping turbulence by downward migration (Härkönen, Pekcan‐Hekim, Hellèn, Ojala, & Horppila, 2014; Pringle, 2007). Sand–gravel bottom was used to minimize the effects of turbulence on water turbidity through sediment resuspension, as the turbulence required for resuspension of sand and gravel is much higher than for small particles of a clayish bottom (e.g., Peterson, 1999). A thin organic layer was allowed to better mimic natural circumstances. Large organic particles are less prone to resuspension than small inorganic ones (Rosa, 1985). The ponds were filled with water led from Lake Syrjänalunen, filtered through a 50‐μm net, 4 weeks prior to the experiments. Water was taken from Lake Syrjänalunen to minimize the effects of turbidity‐causing suspended particles and light‐absorbing humic substances. Lake Syrjänalunen is a groundwater lake thus having clear water with low color (5–10 mg Pt/L) and turbidity (<2 FNU) (Arvola et al., 2009; Hertta database 2015; Horppila, Estlander, 2010). Six ponds were randomly assigned to calm or turbulent treatments with three replicates each. Four sequential experiments were conducted, resulting in 12 replicates per treatment.

### 2.2 Prey and predators

To have a diverse zooplankton community for the experiments, zooplankton was hauled (150‐μm plankton net, diameter 50 cm) from the epilimnion of Lake Iso Valkjärvi and Lake Majajärvi nearby the field station (lake descriptions: Estlander, Nurminen, Olin, Vinni, & Horppila, 2009; Horppila, Olin, 2010). Two weeks before the start of the experiments, an equal mixture of Lake Majajärvi and Lake Iso Valkjärvi zooplankton (corresponding to 1400 L of lake water) was added to each pond. Benthic macroinvertebrates such as various insect larvae were allowed to colonize the ponds for 4 weeks prior to the start of the experiments. In addition, 120 individuals of the isopod *Asellus aquaticus* L. were added to each mesocosm, corresponding to densities found in the nearby lakes.

Perch and roach were caught from Lake Majajärvi using trap nets and were acclimatized for 3 weeks before the experiments in ponds similar to the ones used in the experiments. The perch used in the experiments had a mean length of 9.8 ± 0.2 cm and weight of 8.8 ± 0.5 g, and the mean length of roach was 10.8 ± 0.4 cm and weight 11.6 ± 0.7 g. Mean length and weight of fish did not differ significantly between treatments for either of the species (ANOVA, ln‐transformed data: perch length, *F*_1,70_ = 0.30, *P *=* *.5868; perch weight, *F*_1,70_ = 1.19, *P *=* *.2786; roach length, *F*_1,67_ = 0.08, *P *=* *.7850; roach weight *F*_1,67_ = 0.07, *P *=* *.7983). Perch and roach of this size are usually mainly planktivorous but feed also on benthic food when available (Horppila et al., 2000; Persson & Greenberg, 1990). Each mesocosm was stocked with three perch and three roach (starved 48 hr prior to experiments), as a 1:1 ratio facilitates testing for competitive asymmetries (Persson, 1987).

The fish and *A. aquaticus* were collected from Lake Majajärvi with permission of the Finnish National Board of Forestry (Metsähallitus, Permit Number: 31875). No endangered species were involved in the study. Ethical concerns on the care and use of experimental animals were followed under permission approved by the Finnish Animal Welfare Commission (Permit Number: STH188A).

### 2.3 Environmental conditions

Submerged artificial plants, mimicking *Elodea canadensis* and *Myriophyllum*, added structural complexity to the mesocosm ponds. The coverage of the plants in each pond was 5%, in line with the circumstances in the local lakes (Estlander et al., 2009). Turbulence was created using four computer‐controlled submersible pumps (Tunze Turbelle Nanostream; Tunze Aquarientechnik GmbH, Penzberg, Germany) placed on the sides of each pond with turbulence treatment (Härkönen et al., 2014; Pekcan‐Hekim et al., 2013). Turbulence measurements were made using an acoustic Doppler velocimeter (ADV) (10 MHz ADVField; Sontek/YSI, San Diego, CA, USA). A 25‐Hz measurement for a period of 2 min was conducted from the middle of the water column from nine different locations randomly distributed in the mesocosms. The root‐mean‐square (RMS) velocities (cm/s) were calculated:

$$\text{RMS}=\sqrt{u_{{\mathrm{rms}}_{x}}^{2}+u_{{\mathrm{rms}}_{y}}^{2}+u_{{\mathrm{rms}}_{z}}^{2}},\;\text{where}\;u_{{\mathrm{rms}}_{x}}=\sqrt{\frac{\sum u_{x}^{2}-{\left(\sum u_{x}\right)}^{2}/n}{n-1}}$$

which is the fluctuation of the flow for Cartesian vector x, and *n* is the number of samples in a 2‐min measurement. The RMS velocities were expressed as averages for the whole pond. The energy dissipation rate (m^2^/s^3^) was calculated for the average RMS velocities

$$\varepsilon =A_{1}\frac{{\text{RMS}}^{3}}{l}$$

where *A*_1_ is a nondimensional constant of order 1 (Kundu & Cohen, 2010; Moum, 1996) and *l* is the water depth (m) which describes the size of the largest vortices. The Reynolds (Re) number (the ratio of inertial forces to viscous forces) was calculated (Peters & Redondo, 1997):

$$\text{Re}=\frac{\text{RMSl}}{v}$$

where *v* is the kinematic viscosity for water (10^−6^ m^2^/s).

The average RMS velocity in the turbulent mesocosms was adjusted to a level of 2.8 cm/s which corresponds to a dissipation rate of 10^−4^ m^2^/s^3^ and Reynolds number 11,200. This level can be observed in shallow lake waters at moderate wind speeds of 4–8 m/s (Baranyai et al., 2011; G.‐Tóth et al., 2011). Such turbulence should affect plankton movement and encounter rates, but should not dislodge zoobenthic animals from their habitats or considerably affect water quality via sediment resuspension. This was also confirmed in preliminary measurements, where in addition to turbulence measurements, the dependence of signal‐to‐noise ratios (SNRs) of the turbulence meter on water turbulence (RMS velocity 0.1–8 cm/s) was studied with regression analysis. SNR values describe the density of particles in the water and thus well indicate water turbidity (Salehi & Strom, 2011). No dependence between turbulence and SNR values was observed (*F*_1,22_ = 0.0037, *R* ^2^ = .0001, *P *=* *.9518).

### 2.4 Sampling and sample analyses

The experiments started at 6 p.m. and ended the next morning at 8 a.m. The perch in Evo district are known to have a diel activity at 6–8 p.m. and 6–8 a.m. (Rask, 1986). Roach are known to be active also at night (Estlander, 2011; Jacobsen & Berg, 1998). Thus, the duration and time period of the experiments covered activity peaks for both fish species. Temperature, pH, color, turbidity, and light intensity were kept equal in the different ponds. To confirm this, at the beginning and at the end of each experiment, water temperature, dissolved oxygen, and pH were measured from the mid‐depth of each pond (YSI 6600V2 sonde; YSI Inc., Yellow Springs, OH, USA). Light intensity in each pond was determined in the beginning and at the end of each replicate experiment with a LI‐192SA quantum sensor (LI‐COR Biosciences, Lincoln, NE, USA) equipped with a LI‐1400 datalogger. Light attenuation coefficients in different treatments were calculated from light intensity measurements at the surface (1 cm below the surface) and at 35 cm depth (e.g., Scheffer, 1998).

Initial experimental zooplankton densities and community structures were sampled with a tube sampler (5.4 cm diameter, 50 cm height) from five random places in each mesocosm (total sample volume 6 L per mesocosm). The samples were filtered through a plankton net (50 μm mesh size) and preserved in 4% formaldehyde. Zooplankton samples were analyzed by inverted microscopy (Olympus CK40; 125× magnification) and identified to species or genus level. From each crustacean taxon, 30 individuals were measured. *Daphnia* sp. were measured from the center of the eye to the base of the tail spine and other species from the anterior edge to the posterior edge of the carapace. Zooplankton biomasses were calculated from individual lengths using length–weight regressions (Bottrell et al., 1976; Culver, Boucherie, Bean, & Fletcher, 1985; Rosen, 1981). The analyses focused on crustacean zooplankton, because smaller zooplankton such as rotifers are usually not included in the food of perch and roach having a length of 10–11 cm (Horppila et al., 2000; Persson & Greenberg, 1990). The macroinvertebrates were sampled before each experiment (three replicate samples per pond) with a tube sampler (diameter 70 mm), sieved through a 0.5‐mm net and preserved frozen. The samples were picked under a stereo microscope and identified to an appropriate level. The wet weight (ww) of each measured taxon was measured with an accuracy of 0.0001 g.

Each experiment was ended by removing the fish from the ponds by electrofishing (Schriver, Bøgestrand, Jeppesen, & Søndergaard, 1995). The fish were measured for total length and weight. The stomach content of perch was analyzed for fullness (scale 0–10) and volume proportions of different food items (Windell, 1971). Roach lacks a distinct stomach, and thus, the content of the anterior third of the gut was analyzed (Vøllestad, 1985). The gut contents were estimated for volume proportions of different food items.

The Levin measure of niche breadth (B) of perch and roach in each pond was calculated with the equation (e.g., Marshall & Elliott, 1997).

$$B=\frac{1}{\Sigma p_{j}^{2}}$$

where *p*_*j*_ = proportion of the diet comprising prey species *j*. The diet overlap between perch and roach in each pond was calculated using Schoener's similarity index (Kahl & Radke, 2006; Schoener, 1970).

$$S=1-0.5(\Sigma_{i=1}^{n}|p_{xi}-p_{yi}|)$$

where *p*_*xi*_ = proportion of food category i in the diet of species *x*

*p*_*yi*_ = proportion of food category i in the diet of species *y*

*n* = number of food categories.

The overlap index was calculated considering all food items and also separately for planktonic and benthic food. Moreover, to study the effects of turbulence on individual level, the population‐wide prevalence of individual specialization (IS) was calculated (Bolnick, Yang, Fordyce, Davis, & Svanbäck, 2002; Svanbäck & Bolnick, 2007). This was done by calculating the proportional similarity index (PS), that is, the diet overlap between an individual *i* and the population (Bolnick et al., 2002; Feinsinger, Spears, & Poole, 1981)

$${\text{PS}}_{i}=1-0.5\sum_{j}\left|p_{ij}-q_{j}\right|$$

where *p*_*ij*_ is the proportion of the *j*th resource category in individual *i*'s diet and *q*_*j*_ is the proportion of the *j*th resource in the populations' niche. For an individual that consumes resources in the same proportion as the population as a whole, the index gets a value of 1, and values approaching 0 indicate high individual variation. IS was measured as the average of individuals' PS values (Bolnick et al., 2002).

### 2.5 Statistical analyses

The possible variations in the availability of different food categories were studied (after confirming the homogeneity of variances with Levene's test, *P *>* *.05 for all taxa) by comparing their initial densities and biomasses in calm and turbulent ponds with one‐way analysis of variance (ANOVA) (ln(*x* + 1)‐transformed data). Two‐way ANOVA was used to analyze the effect of fish species and water turbulence on the proportion of different food categories in the diet (arcsin $\sqrt{x}$‐transformed data), niche breadth, and prevalence of individual variation. The effects of water turbulence on diet overlap between perch and roach were analyzed with one‐way ANOVA. Additionally, the effects of turbulence on water temperature, pH, oxygen concentration, and light extinction were analyzed with one‐way ANOVA (ln(*x* + 1)‐transformed data). In order to consider potential difference in effects between experimental days, as well as to compensate dfs according to the repeated experimental design, factor “experiment day” was included as a blocking random factor to the two‐way ANOVA, and in the further analysis, we dropped experiment day out because it was not significant.

## 3 Results

### 3.1 Water quality

The average water temperature was 15.8°C in the calm ponds and 15.9°C in the turbulent ponds with no difference between the treatments (*F*_1,46_ = 0.03, *P *=* *.8575). The average concentration of dissolved oxygen was 9.7 mg/L (minimum 8.6 mg/L) in both treatments with no treatment effect (*F*_1,46_ = 0.04, *P *=* *.8361). Water pH was on average 7.2 in both calm and turbulent ponds, with no differences between treatments (*F*_1,46_ = 0.00, *P *=* *.9982). Light attenuation coefficient was 0.009/cm in both calm and turbulent ponds (*F*_1,46_ = 0.01, *P *=* *.9271).

### 3.2 Availability of food

The most abundant cladocerans in the ponds were *Bosmina* sp. (mainly *B. coregoni* Baird and *B. longirostris* O. F Müller) that had a biomass of 30.8 μg/L C (density 39.2 ind./L) in the calm ponds and 37.7 μg/L C (52.2 ind./L) in the turbulent ponds (Table 1; Figure 1). The biomass of *Daphnia* sp. (mainly *D. cristata* Sars, *D. cucullata* Sars) remained <10/L C. The cladocerans *Holopedium gibberum* Zaddach, *Scapholeberis mucronata* O. F Müller, *Polyphemus pediculus* L., and *Ceriodaphnia* sp. had biomasses <1/L C in both treatments. Copepods had an average biomass of 53.2/L C in the calm ponds and 51.5/L C in the turbulent ponds. No statistical between‐treatment differences in the initial biomass or density were detected in any of the zooplankton taxa (Table 1).

**Table 1:** The initial abundance of zooplankton (density in ind./L, biomass in μg/L C) (± 95% confidence limits) and benthic macroinvertebrates (density in ind./m^2^, biomass in g/m^2^ ww). Results of the one‐way ANOVA on the between‐treatment differences (calm vs. turbulent ponds) in the initial density and biomass of the various taxa

	Calm	Turbulent	*F*_1,22_	*P*
Density				
*Bosmina*	39.2 ± 21.7	52.2 ± 22.6	0.64	.4334
*Daphnia*	2.6 ± 1.7	8.3 ± 5.7	2.65	.1175
Other cladocera	3.2 ± 3.7	2.3 ± 1.5	0.02	.8919
Cyclopoida	4.9 ± 1.6	9.3 ± 4.4	1.63	.2155
Calanoida	4.4 ± 1.3	4.0 ± 1.5	0.66	.4242
Chironomidae	2641.8 ± 1494.7	2944.9 ± 1666.2	0.00	.9811
Other benthos	72.2 ± 40.8	43.3 ± 24.5	0.20	.6578
Biomass				
*Bosmina*	30.8 ± 13.0	37.7 ± 15.6	0.38	.5431
*Daphnia*	2.3 ± 1.5	8.1 ± 5.3	3.56	.0724
Other cladocera	6.0 ± 6.6	2.6 ± 2.0	0.21	.6527
Cyclopoida	12.8 ± 5.5	27.3 ± 16.4	1.65	.2123
Calanoida	40.4 ± 14.9	24.2 ± 8.4	1.32	.2634
Chironomidae	4.3 ± 2.4	4.9 ± 2.8	0.20	.6573
Other benthos	0.8 ± 0.44	0.2 ± 0.10	1.27	.2711

The average density of benthic macroinvertebrates was 2,707 ind./m^2^ in the calm ponds and 2,988 ind./m^2^ in the turbulent ponds (Table 1). The average biomass was 5.1 g ww/m^2^ in both treatments. Chironomid larvae were the dominant group, making up 85% of the macroinvertebrate biomass in the calm ponds and 96% in the turbulent ponds. The rest of the macroinvertebrate biomass consisted of various taxa including, for example, *Asellus aquaticus* and Oligochaeta. No statistical between‐treatment differences in the availability of any zoobenthic taxa were detected (Table 1).

### 3.3 Diet composition and treatment effects

**Figure 2:**
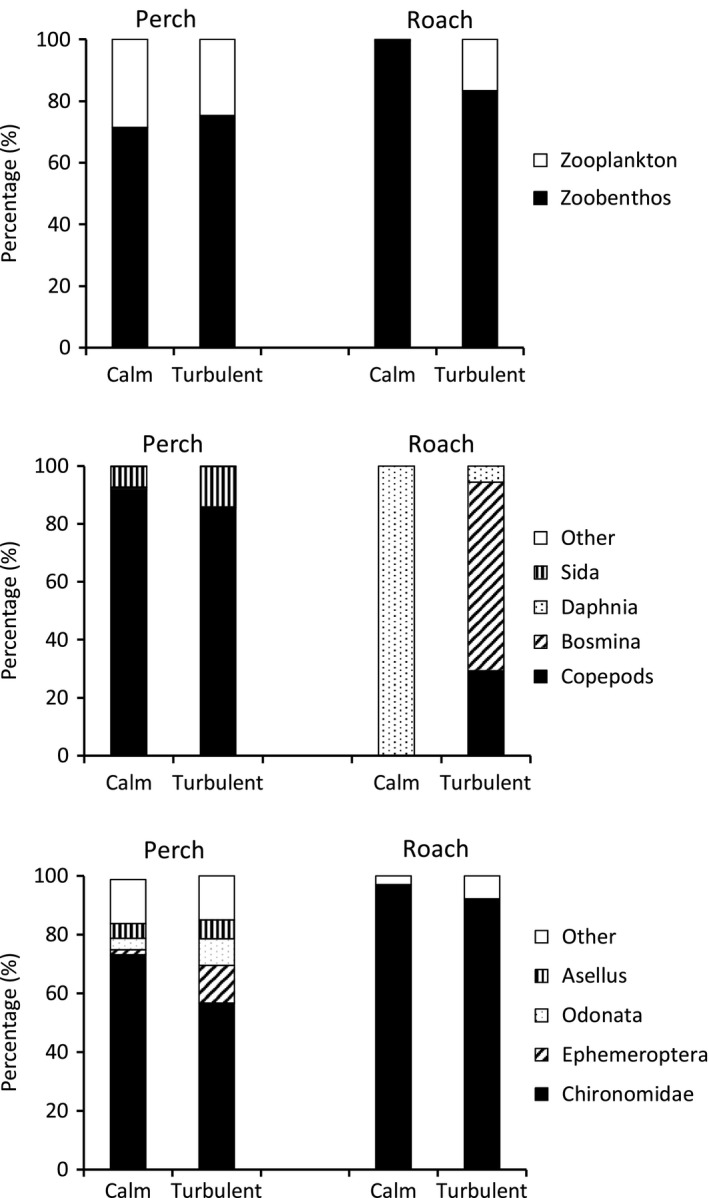
The percentage food composition of perch and roach in the calm and turbulent conditions. Top: relationship of planktonic and benthic food. Middle: composition of planktonic food. Bottom: composition of benthic food

The diet of both fish species consisted mainly of benthic macroinvertebrates in both treatments, but in the diet of roach the proportion was significantly higher than in the diet of perch (Figure 2; Table 2). In perch, macroinvertebrates made up 72% of the stomach contents in the calm ponds and 75% in the turbulent ponds (Figure 2). For roach, the proportion was 99% in calm ponds and 78% in the turbulent ponds. Due to the stronger effect of turbulence on the proportion of benthic food in roach diet, the turbulence **×** fish species interaction was statistically significant (Table 2). The most common benthic macroinvertebrates in perch diet were chironomid larvae: 72% of the benthic food in calm ponds and 57% in the turbulent ponds (Figure 2). Within the macroinvertebrate diet, the proportion of Chironomidae decreased significantly in turbulence for both fish species (Figure 2; Table 2). In perch, this did not affect the total proportion of macroinvertebrates, because Ephemeroptera had a significantly higher diet proportion in turbulent ponds (12%) than in calm ponds (2%). Also, the proportion of Odonata and *Asellus* was elevated in perch diets in the turbulent compared with calm ponds.

**Table 2:** Results of the two‐way ANOVA on the effects of water turbulence (calm vs. turbulent) and fish species (perch vs. roach) on the proportion of different food categories in the diet. Statistically significant effects are bolded

	Turbulence		Fish species		Interaction	
	*F*	*P*	*F*	*P*	*F*	*P*
Zooplankton	0.32	.5744	43.85	**<.0001**	0.25	.6154
Cladocera	6.27	**.0136**	0.56	.4544	3.58	.0608
*Bosmina* sp.	5.80	**.0176**	6.46	**.0123**	9.29	**.0028**
*Daphnia* sp.	0.58	.4485	3.97	**.0485**	0.23	.6342
*Sida crystallina*	1.12	.2925	9.82	**.0022**	0.93	.3380
Copepoda	0.00	.9656	44.12	**<.0001**	1.50	.2224
Benthic macroinv.	2.01	.1589	21.49	**<.0001**	4.52	**.0356**
Chironomidae	5.21	**.0241**	83.55	**<.0001**	0.51	.4766
Ephemeroptera	7.26	**.0081**	11.81	**.0008**	5.83	**.0172**
Odonata	3.37	.0687	16.71	**<.0001**	2.76	.0994
*Asellus*	0.33	.5687	13.04	**.0004**	0.29	.5933

The proportion of zooplankton in the diets of perch (26%) was on average higher than in those of roach (9%). Zooplanktonic diet of perch was dominated by copepods in both treatments, while cladoceran food consisted mainly of *Sida crystallina* and *Bosmina* (Figure 2). The proportion of *Sida* increased and the proportion of *Bosmina* decreased in turbulence. In roach guts, only *Daphnia* were found in the calm ponds, while *Bosmina* dominated the diets in turbulent ponds. Copepods were found in roach guts only in the turbulent treatments. The proportion of *Bosmina* was significantly affected by the fish species, by turbulence, and by turbulence **×** species interaction (Table 2).

The fish were feeding actively with no differences between the treatments. In perch, one fish had an empty stomach in both treatments. The average stomach fullness was 7.7 for calm ponds and 8.5 for turbulent ponds. In roach, empty guts were found in eight fish in the calm ponds and seven fish in the turbulent ponds. Two roach were not recovered.

### 3.4 Frequency of occurrence of different food categories

Chironomids were included in the diet of almost all perch, in both the calm (94%) and turbulent (97%) ponds (Table 3). Copepods were eaten by >70% of the perch individuals in both treatments. The frequency of occurrence of Ephemeroptera, Odonata, and *Sida crystallina* in the diets of perch was higher in turbulent than in calm ponds, while the occurrence of *Bosmina* decreased in turbulent ponds (Table 3). All roach fed on chironomids in both treatments. *Bosmina* and copepods were not eaten by a single roach in the calm bonds, but in turbulence *Bosmina* was consumed by 26% and copepods by 11% of the roach (Table 3).

**Table 3:** The frequency of occurrence (% of fish having the food category in the diet) of the most important food categories in the food of perch and roach in the calm and turbulent ponds

	Perch		Roach	
	Calm	Turbulent	Calm	Turbulent
*Bosmina*	11	6	0	26
*Daphnia*	0	3	7	7
Copepoda	71	76	0	11
*Sida crystallina*	17	30	0	0
Chironomidae	97	94	100	100
Ephemeroptera	9	42	0	0
Odonata	17	36	0	0

### 3.5 Diet overlap, niche breadth, and individual specialization

**Figure 3:**
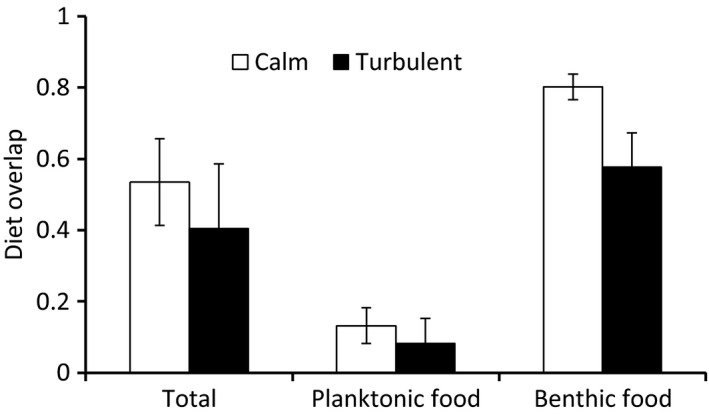
The diet overlap (Schoener's index) of perch and roach in calm and turbulent conditions (±95% confidence intervals)

The diet overlap between perch and roach was partly affected by turbulence (Figure 3). Diet overlap for benthic food was significantly decreased in turbulence conditions (*F*_1,21_ = 8.30, *P *=* *.009), while there were no effects of turbulence on diet overlap when considering all food categories (*F*_1,21_ = 1.88, *P *=* *.1845) or planktonic diet (*F*_1,5_ = 0.29, *P *=* *.6174).

**Figure 4:**
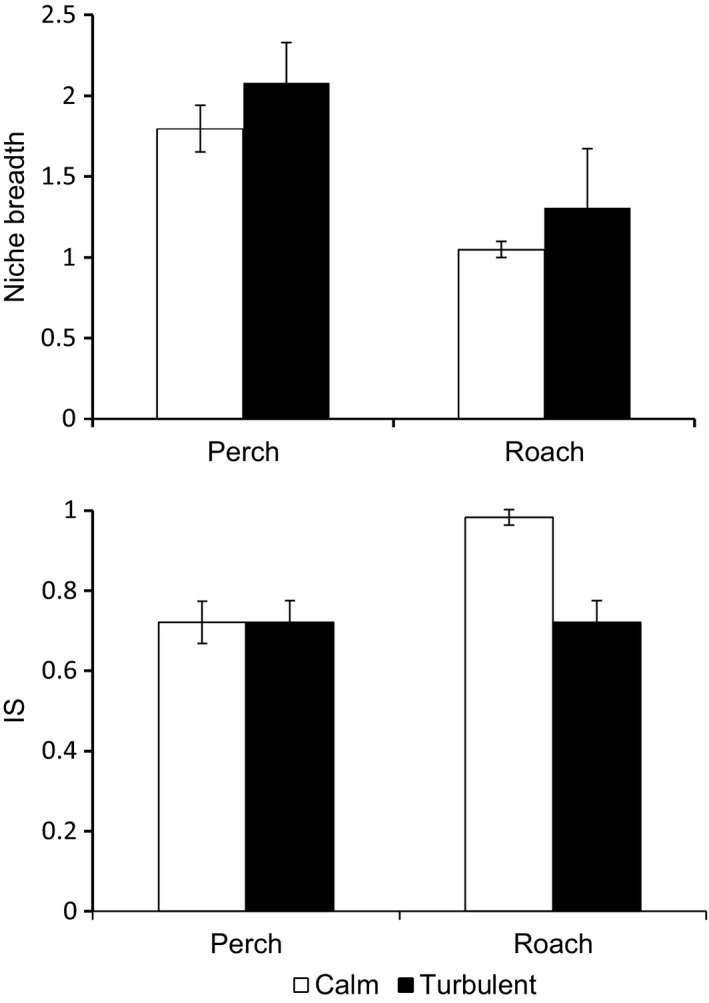
The niche breadth (Levins's index, top) and prevalence of individual specialization (bottom) of perch and roach in calm and turbulent conditions (±95% confidence intervals)

Perch had larger niche breadth than roach (*F*_1,1_ = 51.30, *P *<* *.0001), and the niche breadth increased for both perch and roach in turbulent water (*F*_1,1_ = 5.88, *P *=* *.0196), with no between‐species difference in the effect of turbulence (turbulence **×** fish species interaction, *F*_1,1_ = 0.12, *P *=* *.7271) (Figure 4). The proportional similarity index (PS) of perch was not affected by turbulence (*F*_1,68_ = 0.07, *P *=* *.7899) and the IS value was 0.72 in both calm and turbulent ponds (Figure 4). In roach, IS values were significantly lower in the turbulent (0.73) than in the calm ponds (0.98) (*F*_1,53_ = 11.97, *P *=* *.0011). Consequently, the turbulence **×** fish species interaction term was highly significant for PS (*F*_1,1_ = 8.42, *P *=* *.0044).

## 4 Discussion

The niche breadth and diet overlap are related to competition intensity (Holbrook & Schmitt, 1989; Robinson & Wilson, 1998; Svanbäck & Bolnick, 2007). Because niche breadth and diet overlap were both affected by turbulence, our results demonstrated that water turbulence affects competition between fish species. We show that turbulence can introduce niche partitioning and allow for the coexistence of species.

Perch and roach both showed typical generalist feeding habits, consuming benthic as well as planktonic food (Bergman, 1990; Horppila et al., 2000; Kahl & Radke, 2006; Persson, 1983). Chironomid larvae dominated the diet of both fish species in calm and turbulent water, probably due to high chironomid abundance and shallow water, which facilitated access to benthic resources. Fish commonly select benthic before planktonic prey due to their larger size and higher energy content. If two consumer species share a preference for one prey type, they may however differ in preference for lower‐ranking prey types (Robinson & Wilson, 1998). This may occur when the preferred prey is highly abundant and easy to use while the consumption of other prey requires specialization (Robinson & Wilson, 1998).

In the calm circumstances, the second‐ranked prey were chironomid pupae for roach (3% of gut contents) and copepods for perch (26% of stomach contents). The clearly higher proportion of zooplankton in the diet of perch was somewhat unexpected as perch is considered a superior forager over roach on benthic food (Persson & Greenberg, 1990). The reason can be found in the zooplankton community structure and the different specialization of the two species. Roach can outcompete perch in capturing cladocerans, but perch may be a more efficient forager on evasively swimming copepods (Lessmark, 1983; Peterka & Matĕna, 2009). As shown in the calm ponds, roach selects for *Daphnia* cladocerans (Estlander et al., 2010; Kahl & Radke, 2006; Peterka & Matĕna, 2009), although the crustacean zooplankton community was dominated by *Bosmina* in density and copepods in biomass. Perch, on the other hand, showed positive selection for copepods which has been connected to the movement conspicuousness of copepods (Mills, Wizzowski, & Jones, 1987; Peterka & Matĕna, 2009; Vašek, Kubečka, Matĕna, & Seda, 2006). Copepods in the ponds were also larger (average length 0.65 mm) than cladocerans (*Daphnia* 0.56 mm, *Bosmina* 0.31 mm) that were generally too small for perch. For a fish of 7–8 cm length, the minimum prey size is c. 0.5 mm for perch and 0.3 mm for roach (Lessmark, 1983). Additionally, copepods are preferred prey as their handling time is low compared to cladocerans (Graeb, Dettmers, Wahl, & Cáceres, 2004).

When turbulence was included, the response by perch differed from roach in terms of overall selectivity for planktonic prey and preference between planktonic and benthic food and in habitat choice. Contrary to our hypothesis, the planktivorous feeding was more affected by turbulence in roach than perch. With turbulence, the proportion of zooplankton in the diet of roach increased, indicating an increase in the use of the pelagic habitat by roach or in zooplankton density in the proximity of the benthic habitat. In contrast to calm conditions, roach discarded *Daphnia* and selected for *Bosmina* and copepods. With a constant predator speed, an increase in prey speed should result in increased encounter rate (Gerritsen & Strickler, 1977; Rothschild & Osborn, 1988). Because the turbulence in the ponds was not strong enough to affect the swimming of fish, but strong enough to affect zooplankton distribution, encounter rates between roach and *Bosmina* likely increased. Roach selectivity decreases with increasing densities of zooplankton (Maszczyk & Gliwicz, 2014), which together with the increased encounter rate led to increased consumption of *Bosmina*. Moreover, as turbulence disturbs evasive responses in copepods (Härkönen et al., 2014; Saiz & Alcaraz, 1992), roach foraging on copepods was enhanced in turbulent water. It must also be acknowledged that prey were not replaced; thus, prey density and selection may have varied during the experiments. However, both species faced the same prey densities; thus, the possible variation does not affect the ultimate conclusions on the species‐specific responses to turbulence.

The digestion rate during the experiments cannot be accurately determined as it depends on multiple factors such as consumption rate and food type (Hofer, Forstner, & Rettenwander, 1982; Persson, 1981). Based on the species‐specific gut passage times and gastric evacuation rates in the experimental temperature (Hofer et al., 1982; Persson, 1981, 1982), it took approximately 7–10 hr for the food to be evacuated. Thus, some consumed food items may not have been observed from fish captured at the end of the experiment. This would however affect the comparison only if food quality showed considerable diurnal variation and if this variation was dependent on turbulence. The similar values of stomach fullness and frequency of empty guts in calm and turbulent ponds suggested however that such phenomena did not take place. The high values of stomach fullness also suggested that most food items were discovered.

Perch did not shift to or from benthic macroinvertebrates or zooplankton between calm and turbulent water, suggesting that perch did not shift habitat vertically. Within food categories, however, the proportions changed. The proportion of chironomids was lower in turbulence, while the proportion of other benthos, mostly Ephemeroptera and Odonata, increased in the turbulence treatment. As these prey prefer structurally complex vegetation‐rich areas (Engblom, 1996; Norling & Sahlén, 1997), this indicates that perch switched to feed more among the macrophytes under turbulent conditions. Additionally, the proportion of copepods decreased and the proportion of the plant‐associated cladoceran *Sida crystallina* increased in the diet of perch under turbulence, which also suggests that a shift to littoral feeding in the turbulent environment took place. The driving factor behind this altered behavior was probably the altered foraging behavior of roach. When perch and roach compete for zooplankton, roach can force perch to shift from zooplanktivory to consuming benthic food (Bergman & Greenberg, 1994; Persson & Greenberg, 1990). Increasing roach density in the pelagic zone may hereby induce a habitat shift in perch (Persson, 1987; Persson & Greenberg, 1990). In calm water, roach fed almost solely on chironomids, leaving planktonic prey to perch, whereas in turbulent conditions, roach increased preying on zooplankton, forcing perch to switch habitat and food source. This behavioral shift in perch was, however, an indirect response to foraging competition, as roach increased consumption of *Bosmina* and not the copepods mainly eaten by perch. Thus, the mere presence of roach disturbed perch. This is in concordance with the findings by Nurminen, Estlander, Olin, and Lehtonen (2014), who showed that feeding rate by planktivorous perch decreased in the presence of roach, while perch had no effect on the feeding rate of roach. Similarly, Persson (1987) demonstrated that the food utilization by perch changed in the presence of roach. It is from these and the present findings intelligible that perch chose the vegetated littoral environment under turbulent conditions. Compared with roach, perch are known to be efficient feeders in structural complexity (Diehl, 1988), and by foraging among macrophytes, they could decrease interspecific competition, resulting in the significant decrease in benthic diet overlap. The effects of turbulence were not tested using single‐species shoals, but previous studies have shown that the response to environmental disturbances does not necessarily depend on the number of species present. Nurminen et al. (2014) showed that the response of perch to disturbance (increasing water color) was similar in pure perch shoals and in shoals together with roach.

Turbulence could negatively affect prey detection in near‐bottom layers by increasing sediment resuspension and water turbidity (Lind, 2003; Nurminen, Pekcan‐Hekim, & Horppila, 2010). However, no differences in water quality between turbulent and calm ponds were detected confirming that the diet changes were not attributed to such turbulence‐mediated changes in water quality. The hydraulic stress caused by turbulence can also disturb the swimming of fish, but this holds only for much stronger turbulence and deeper‐bodied fish species (Gabel, Stoll, Fischer, Pusch, & Garcia, 2011). Thus, turbulence probably did not disturb the benthic feeding of roach but enhanced their zooplanktivory, which resulted in decreased diet proportion of benthic food. This conclusion was supported also by the fact that the frequency of occurrence of macroinvertebrates in the diets of roach was not affected by turbulence, whereas the frequency of occurrence of cladocerans and copepods increased in turbulent ponds. However, the increase in their individual specialization revealed that only a fraction of the roach turned to zooplanktivory. Both the average volume proportion and frequency of occurrence of zooplankton in roach diets increased c. 20% suggesting that one‐fifth of the roach shifted foraging habits in turbulence, and these individuals concentrated mainly on *Bosmina*. Such specialization is beneficial from an individual point of view, because chironomids and zooplankton require a very different handling technique, which makes switching between prey difficult (Persson, 1985). Accordingly, it has been shown earlier that in shoals of roach, the most active individuals swimming in the front have a higher tendency to feed on plankton, while the more passive fish concentrate on benthic food (Krause, 1993). In perch, the IS value was at a lower level also in the calm conditions. This was in line with findings that perch form looser schools and show larger variation in individual feeding compared to roach (Christensen & Persson, 1993; Nurminen et al., 2010). Shoals of perch also include considerable individual variation in the ability to learn to utilize novel food resources (Magnhagen & Staffan, 2003).

Turbulence showed species‐specific effects by partitioning foraging niches and decreasing interspecific competition. The study also demonstrated that turbulence can affect habitat use of competing species by affecting prey choice to the point of habitat shift (Werner & Hall, 1979). The results supported the view that roach can dominate the pelagic habitat over perch (Persson & Greenberg, 1990), but also indicated that the superiority of roach over perch in planktivorous feeding is not as straightforward as often presented, and may depend on turbulence (cf. Peterka & Matĕna, 2009, 2011). In calm water, perch can be the main planktivore even in the presence of roach, if the availability of zoobenthos is high and roach concentrates on benthic food. The results suggested that dominance of roach in the pelagic habitat and individual variation in their foraging behavior increase with turbulence. The response of fish to turbulence thus depends both on their prey searching strategy and on the turbulence‐induced changes in the accessibility of different food resources. Contrary to previous results, the results suggested that cruise predators may benefit from turbulence more than pause‐travel predators if turbulence widens their food spectrum. More studies with varying fish densities and food resources are needed, but the results indicated that the combined effects of water turbulence, feeding strategy of fish, and zooplankton community structure can determine the competitive relationships among fish communities. Turbulence is regulated by wind velocity or water level, and in many lake ecosystems, both of these factors are changing together with climate. Therefore, climate change may have considerable effects on lacustrine fish communities and should be taken into a close consideration.

## Conflict of Interest

None declared.

## Data Accessibility

Data will be available from the DataDryad database.

## References

[ece32593-bib-0001] Amarasekare, P. (2003). Competitive coexistence in spatially structured environments: A synthesis. Ecology Letters, 6, 1109–1122.

[ece32593-bib-0002] Arvola, L. , Rask, M. , Ruuhijärvi, J. , Tulonen, T. , Vuorenmaa, J. , Ruoho‐Airola, T. , & Tulonen, J. (2009). Long‐term patterns on pH and colour in small acidic boreal lakes of varying hydrological landscape settings. Biogeochemistry, 101, 269–279.

[ece32593-bib-0003] Baranyai, E. , G.‐Toth, L. , Vári, A. , & Homonnay, Z. G. (2011). The effect of turbulent intensities on the distribution of zooplankton in the shallow, large lake Balaton (Hungary). Knowledge and Management of Aquatic Ecosystems, 400, 07.

[ece32593-bib-0004] Bergman, E. (1990). Effects of roach *Rutilus rutilus* on two percids, *Perca fluviatilis* and *Gymnocephalus cernua*: Importance of species interactions for diet shifts. Oikos, 57, 241–249.

[ece32593-bib-0005] Bergman, E. , & Greenberg, L. (1994). Competition between a planktivore, a benthivore and a species with ontogenic diet shifts. Ecology, 75, 1233–1245.

[ece32593-bib-0006] Bolnick, D. I. , Yang, L. H. , Fordyce, J. A. , Davis, J. M. , & Svanbäck, R. (2002). Measuring individual‐level resource specialization. Ecology, 83, 2936–2941.

[ece32593-bib-0007] Bottrell, H. H. , Duncan, A. , Gliwicz, Z. , Grykierek, E. , Herzig, A. , Hillbricht‐Ilkowska, A. , … Weglenska, T. (1976). A review of some problems in zooplankton production studies. Norwegian Journal of Zoology, 24, 419–456.

[ece32593-bib-0009] Chesson, P. (2000). General theory of competitive coexistence in spatially‐varying environments. Theoretical Population Biology, 58, 211–237. 11120650 10.1006/tpbi.2000.1486

[ece32593-bib-0010] Christensen, B. , & Persson, L. (1993). Species‐specific antipredatory behaviours: Effect of prey choice in different habitats. Behavioural Ecology and Sociobiology, 32, 1–9.

[ece32593-bib-0011] Conradt, L. , Clutton‐Brock, T. H. , & Thomson, D. (1999). Habitat segregation in ungulates: Are males forced into suboptimal foraging habitats through indirect competition by females? Oecologia, 119, 367–377. 10.1007/s004420050797 28307759

[ece32593-bib-0012] Culver, D. A. , Boucherie, M. M. , Bean, D. J. , & Fletcher, J. W. (1985). Biomass of freshwater crustacean zooplankton from length‐weight regressions. Canadian Journal of Fisheries and Aquatic Sciences, 42, 1380–1390.

[ece32593-bib-0013] Diehl, S. (1988). Foraging efficiency of three freshwater fishes: Effects of structural complexity and light. Oikos, 53, 207–2014.

[ece32593-bib-0014] Dunham, A. E. (1980). An experimental study of interspecific competition between the iguanid lizards *Scelopus meriami* and *Urasaurus ornatus* . Ecological Monographs, 50, 309–330.

[ece32593-bib-0015] Engblom, E. (1996). Ephemeroptera, Mayflies In Nilsson A. (Ed.), Aquatic insects of North Europe. A taxonomic Handbook. Volume 1 (pp. 13–53). Stenstrup: Apollo Books.

[ece32593-bib-0016] Estlander, S. (2011). Fishes of the Darkness. Water colour‐regulated competitive interactions in humic lakes. Ph. D. Thesis. Department of Environmental Sciences, University of Helsinki.

[ece32593-bib-0017] Estlander, S. , Nurminen, L. , Olin, M. , Vinni, M. , & Horppila, J. (2009). Seasonal fluctuations in macrophyte cover and water transparency of four brown‐water lakes: Implications for crustacean zooplankton in littoral and pelagic habitats. Hydrobiologia, 620, 109–120.

[ece32593-bib-0018] Estlander, S. , Nurminen, L. , Olin, M. , Vinni, M. , Immonen, S. , Rask, M. , … Lehtonen, H. (2010). Diet shifts and food selection of perch (*Perca fluviatilis*) and roach (*Rutilus rutilus* (L.)) in humic lakes of varying water colour. Journal of Fish Biology, 77, 241–256. 20646150 10.1111/j.1095-8649.2010.02682.x

[ece32593-bib-0019] Feinsinger, P. , Spears, E. E. , & Poole, R. W. (1981). A simple measure of niche breadth. Ecology, 62, 27–32.

[ece32593-bib-0020] Ficke, A. D. , Myrick, C. A. , & Hansen, L. J. (2007). Potential impacts of global climate change on freshwater fisheries. Reviews in Fish Biology and Fisheries, 17, 581–613.

[ece32593-bib-0021] Gabel, F. , Stoll, S. , Fischer, P. , Pusch, M. T. , & Garcia, X.‐F. (2011). Waves affect predator‐prey interactions between fish and benthic invertebrates. Oecologia, 165, 101–109. 21104276 10.1007/s00442-010-1841-8

[ece32593-bib-0022] Gause, G. F. (1934). The struggle for existence. Dover Phoenix Editions. Mineola, NY: Dover Publications Inc.

[ece32593-bib-0023] Gerritsen, J. , & Strickler, R. (1977). Encounter probabilities and community structure in zooplankton: A mathematical model. Journal of the Fisheries Research Board of Canada, 34, 73–82.

[ece32593-bib-0024] Graeb, B. D. S. , Dettmers, J. M. , Wahl, D. H. , & Cáceres, C. E. (2004). Fish size and prey availability affect growth, survival, prey selection, and foraging behavior of larval yellow perch. Transactions of the American Fisheries Society, 133, 504–514.

[ece32593-bib-0025] Greene, C. H. (1986). Patterns of prey selection: Implications of predator foraging tactics. American Naturalist, 128, 824–839.

[ece32593-bib-0026] G.‐Tóth, L. , Parpala, L. , Balogh, C. , Tátrai, I. , & Baranyai, E. (2011). Zooplankton community response to enhanced turbulence generated by water‐level decrease in Lake Balaton, the largest shallow lake in Central Europe. Limnology and Oceanography, 56, 2211–2222.

[ece32593-bib-0027] Härkönen, L. , Pekcan‐Hekim, Z. , Hellèn, N. , Ojala, A. , & Horppila, J. (2014). Combined effects of turbulence and different predation regimes on zooplankton in high‐colored water ‐ implications of environmental change for lakes. PlosOne, 9(11), e111942. 10.1371/journal.pone.0111942 PMC4223065 25375952

[ece32593-bib-0028] Hertta database (2015). OIVA Environment and Geographic Information Service. http://www.syke.fi/avointieto. Finnish Environment Institute.

[ece32593-bib-0029] Hofer, R. , Forstner, H. , & Rettenwander, R. (1982). Duration of gut passage and its dependence on temperature and food consumption in roach, *Rutilus rutilus* L: Laboratory and field experiments. Journal of Fish Biology, 20, 289–299.

[ece32593-bib-0030] Holbrook, S. J. , & Schmitt, R. J. (1989). Resource overlap, prey dynamics, and the strength of competition. Ecology, 70, 1943–1953.

[ece32593-bib-0031] Horppila, J. , Estlander, S. , Olin, M. , Pihlajamäki, J. , Vinni, M. , & Nurminen, L. (2010). Gender‐dependent effects of water quality and conspecific density on the feeding rate of fish – factors behind sexual size dimorphism. Oikos, 120, 855–861.

[ece32593-bib-0033] Horppila, J. , Olin, M. , Vinni, M. , Estlander, S. , Nurminen, L. , Rask, M. , … Lehtonen, H. (2010). Perch production in forest lakes: The contribution of abiotic and biotic factors. Ecology of Freshwater Fish, 19, 257–266.

[ece32593-bib-0034] Horppila, J. , Ruuhijärvi, J. , Rask, M. , Karppinen, C. , Nyberg, K. , & Olin, M. (2000). Seasonal changes in the food composition and relative abundance of perch and roach ‐ a comparison between littoral and pelagial zones of a large lake. Journal of Fish Biology, 56, 51–72.

[ece32593-bib-0035] Imboden, D. M. , & Wüest, A. (1995). Mixing mechanisms in lakes In Lerman A., Imboden D. & Gat J. (Eds.), Physics and chemistry of lakes (2nd edn, pp. 83–138). Berlin, Heidelberg, New York: Springer‐Verlag.

[ece32593-bib-0036] Jacobsen, L. , & Berg, S. (1998). Diel variation in habitat use, by planktivores in field enclosure experiments: The effects of submerged macrophytes and predation. Journal of Fish Biology, 53, 1207–1219.

[ece32593-bib-0038] Jeppesen, E. , Søndergaard, M. , & Jensen, J. P. (2003). Climatic warming and regime shifts in lake food webs—some comments. Limnology and Oceanography, 48, 1346–1349.

[ece32593-bib-0039] Joensuu, L. , Pekcan‐Hekim, Z. , Hellèn, N. , & Horppila, J. (2013). Turbulence disturbs vertical refuge use by *Chaoborus flavicans* larvae and increases their horizontal dispersion. Freshwater Biology, 58, 1997–2006.

[ece32593-bib-0040] Jones, M. E. , & Barmuta, L. A. (1998). Diet overlap and relative abundance of sympatric dasyurid carnivores: A hypothesis of competition. Journal of Animal Ecology, 67, 410–421.

[ece32593-bib-0041] Kahl, U. , & Radke, R. J. (2006). Habitat and food resource use of perch and roach in a deep mesotrophic reservoir: Enough space to avoid competition? Ecology of Freshwater Fish, 15, 48–56.

[ece32593-bib-0042] Kiørboe, T. , & Saiz, E. (1995). Planktivorous feeding in calm and turbulent environments: With emphasis on copepods. Marine Ecology Progress Series, 122, 135–145.

[ece32593-bib-0043] Krause, J. (1993). The relationship between foraging and shoal position in a mixed shoal of roach (*Rutilus rutilus*) and chub (*Leuciscus cephalus*): A field study. Oecologia, 93, 356–359. 10.1007/BF00317878 28313435

[ece32593-bib-0044] Kundu, P. K. , & Cohen, I. M. (2010). Fluid mechanics. San Diego, CA: Academic Press.

[ece32593-bib-0045] Lack, D. (1971). Ecological isolation in birds. Cambridge, MA: Harvard University Press.

[ece32593-bib-0046] Lammens, E. H. R. R. , de Nie, H. W. , Vijverberg, J. , & van Densen, W. L. T. (1985). Resource partitioning and niche shifts of bream (*Abramis brama*) and eel (*Anguilla anguilla*) mediated by predation of smelt (*Osmerus eperlanus*) on *Daphnia hyalina* . Canadian Journal of Fisheries and Aquatic Sciences, 42, 1342–1351.

[ece32593-bib-0047] Lee, P.‐Y. , & Suen, P.‐J. (2012). Niche partitioning of fish assemblages in a mountain stream with frequent natural disturbances ‐ an examination of microhabitat in riffle areas. Ecology of Freshwater Fish, 21, 255–265.

[ece32593-bib-0048] Lessmark, O. (1983). Competition between perch (Perca fluviatilis) and roach (Rutilus rutilus) in south Swedish lakes. Ph. D. thesis. Institute of Limnology, University of Lund.

[ece32593-bib-0049] Lind, O. T. (2003). Suspended clay's effect on lake and reservoir limnology. Archiv fur Hydrobiologie. Supplement volumes, Monographic studies, 139, 327–360.

[ece32593-bib-0050] MacKenzie, B. R. , & Kiørboe, T. (1995). Encounter rates and swimming behavior of pause‐travel and cruise larval fish predators in calm and turbulent laboratory environments. Limnology and Oceanography, 40, 1278–1289.

[ece32593-bib-0051] MacKenzie, B. R. , & Leggett, W. C. (1991). Quantifying the contribution of small‐scale turbulence to the encounter rates between larval fish and their zooplankton prey: Effects of wind and tide. Marine Ecology Progress Series, 73, 149–160.

[ece32593-bib-0052] MacKenzie, B. R. , Miller, T. J. , Cyr, S. , & Leggett, W. C. (1994). Evidence for dome‐shaped relationship between turbulence and larval fish ingestion rates. Limnology and Oceanography, 39, 1790–1799.

[ece32593-bib-0053] Magnhagen, C. , & Staffan, F. (2003). Social learning in young‐of‐the‐year perch encountering a novel food type. Journal of Fish Biology, 63, 824–829.

[ece32593-bib-0054] Marshall, S. , & Elliott, M. (1997). A comparison of univariate and multivariate numerical and graphical techniques for determining inter‐ and intraspecific feeding relationships of estuarine fish. Journal of Fish Biology, 51, 526–545.

[ece32593-bib-0055] Maszczyk, P. , & Gliwicz, Z. M. (2014). Selectivity by planktivorous fish at different prey densities, heterogeneities, and spatial scales. Limnology and Oceanography, 59, 68–78.

[ece32593-bib-0056] Mehner, T. , Diekmann, M. , Brämick, U. , & Lemcke, R. (2005). Composition of fish communities in German lakes as related to lake morphology, trophic state, shore structure and human‐use intensity. Freshwater Biology, 50, 70–85.

[ece32593-bib-0057] Mills, E. L. , Wizzowski, D. V. , & Jones, S. R. (1987). Food conditioning and prey selection of young yellow perch (*Perca flavescens*). Canadian Journal of Fisheries and Aquatic Sciences, 44, 549–555.

[ece32593-bib-0058] Moum, J. N. (1996). Energy‐containing scales of turbulence in the ocean thermocline. Journal of Geophysical Research, 191, 14095–14109.

[ece32593-bib-0059] Norling, U. , & Sahlén, G. (1997). Odonata, dragonflies and damselflies In Nilsson A. (Ed.), Aquatic insects of North Europe. A taxonomic handbook. Volume 2 (pp. 13–65). Stenstrup: Apollo Books.

[ece32593-bib-0060] Nurminen, L. , Estlander, S. , Olin, M. , & Lehtonen, H. (2014). Feeding efficiency of planktivores under disturbance, the effect of water colour, predation threat and shoal composition. Journal of Fish Biology, 84, 1195–1201. 24689675 10.1111/jfb.12328

[ece32593-bib-0061] Nurminen, L. , Pekcan‐Hekim, Z. , & Horppila, J. (2010). Feeding efficiency of planktivorous perch (*Perca fluviatilis*) and roach (*Rutilus rutilus*) in varying turbidity – an individual‐based approach. Journal of Fish Biology, 76, 1848–1855. 20557636 10.1111/j.1095-8649.2010.02600.x

[ece32593-bib-0062] Olin, M. , Rask, M. , Ruuhijärvi, J. , Kurkilahti, M. , Ala‐Opas, P. , & Ylönen, O. (2002). Fish community structure in mesotrophic and eutrophic lakes of southern Finland: The relative abundances of percids and cyprinids along a trophic gradient. Journal of Fish Biology, 60, 593–612.

[ece32593-bib-0063] Pekcan‐Hekim, Z. , Joensuu, L. , & Horppila, J. (2013). Predation by a visual planktivore perch (*Perca fluviatilis*) in a turbulent and turbid environment. Canadian Journal of Fisheries and Aquatic Sciences, 70, 854–859.

[ece32593-bib-0064] Persson, L. (1981). The effects of temperature and meal size on the rate of gastric evacuation in perch (*Perca fluviatilis*) fed on fish larvae. Freshwater Biology, 11, 131–138.

[ece32593-bib-0065] Persson, L. (1982). Rate of food evacuation in roach (*Rutilus rutilus*) in relation to temperature, and the application of evacuation rate estimates for studies on the rate of food consumption. Freshwater Biology, 12, 203–210.

[ece32593-bib-0066] Persson, L. (1983). Food consumption and competition between age classes in a perch *Perca fluviatilis* population in a shallow eutrophic lake. Oikos, 40, 197–207.

[ece32593-bib-0067] Persson, L. (1985). Optimal foraging: The difficulty of exploiting different feeding strategies simultaneously. Oecologia, 67, 338–341. 10.1007/BF00384938 28311566

[ece32593-bib-0068] Persson, L. (1986). Temperature‐induced shift in foraging ability in two fish species, roach (*Rutilus rutilus*) and perch (*Perca fluviatilis*): Implications for coexistence between poikilotherms. Journal of Animal Ecology, 55, 829–839.

[ece32593-bib-0069] Persson, L. (1987). Effects of habitat and season on competitive interactions between roach (*Rutilus rutilus*) and perch (*Perca fluviatilis*). Oecologia, 73, 170–177. 10.1007/BF00377504 28312284

[ece32593-bib-0070] Persson, L. , & Greenberg, L. (1990). Juvenile competitive bottlenecks: The perch (*Perca fluviatilis*) – roach (*Rutilus rutilus*) interaction. Ecology, 71, 44–56.

[ece32593-bib-0071] Peterka, J. , & Matĕna, J. (2009). Differences in feeding selectivity and efficiency between youg‐of‐the‐year European perch (*Perca fluviatilis*) and roach (*Rutilus rutilus*) – field observations and laboratory experiments on the importance of prey movement apparency vs. evasiveness. Biologia, 64, 786–794.

[ece32593-bib-0072] Peterka, J. , & Matĕna, J. (2011). Feeding behavior determining differential capture success of evasive prey in underyearling European perch (*Perca fluviatilis* L.) and roach (*Rutilus rutilus* (L.)). Hydrobiologia, 661, 113–121.

[ece32593-bib-0073] Peters, F. , & Redondo, J. M. (1997). Turbulence generation and measurement: Application to studies on plankton. Scientia Marina, 61, 205–228.

[ece32593-bib-0074] Peterson, E. L. (1999). Benthic shear stress and sediment condition. Aquacultural Engineering, 21, 85–111.

[ece32593-bib-0075] Petren, K. , & Case, T. J. (1996). An experimental demonstration of exploitation competition in an ongoing invasion. Ecology, 49, 704–726.

[ece32593-bib-0076] Pianka, E. R. , & Huey, R. B. (1978). Comparative ecology, resource utilization and niche Segregation among gekkonid lizards in the southern Kalahari. Copeia, 1978/4, 691–701.

[ece32593-bib-0077] Pickett, S. T. A. , Kolasa, J. , Arnesto, J. J. , & Collins, S. L. (1989). The ecological concept of disturbance and its expression at various hierarchial levels. Oikos, 54, 129–136.

[ece32593-bib-0078] Pringle, J. M. (2007). Turbulence avoidance and the wind‐driven transport of plankton in the surface Ekman layer. Continental Shelf Research, 27, 670–678.

[ece32593-bib-0079] Rask, M. (1986). The diet and diel feeding activity of perch *Perca fluviatilis* L. in a small lake in southern Finland. Annales Zoologici Fennici, 23, 49–56.

[ece32593-bib-0080] Rask, M. , Appelberg, M. , Beier, U. , Hesthagen, T. , Tammi, J. , & Lappalainen, A. (2000). Fish status survey of Nordic lakes. Species composition, distribution, effects of environmental changes. TemaNord, 508, Nordic Council of Ministers, Copenhagen. ISBN 92‐893‐0422‐7, 60 p.

[ece32593-bib-0081] Rask, M. , Viljanen, M. , & Sarvala, J. (1999). Humic lakes as fish habitats In Keskitalo J., & Eloranta P. (Eds.), Limnology of humic waters (pp. 209–224). Leiden: Backhuys Publishers, ISBN 90‐5782‐029‐3.

[ece32593-bib-0082] Robinson, B. , & Wilson, D. S. (1998). Optimal foraging, specialization, and solution to Liem's paradox. American Naturalist, 151, 223–235. 10.1086/286113 18811353

[ece32593-bib-0083] Rosa, F. (1985). Sedimentation and sediment resuspension in Lake Ontario. Journal of Great lakes Research, 11, 13–25.

[ece32593-bib-0084] Rosen, R. A. (1981). Length‐dry weight relationships of some freshwater zooplankton. Journal of Freshwater Ecology, 1, 225–229.

[ece32593-bib-0085] Rothschild, B. J. , & Osborn, T. R. (1988). Small‐scale turbulence and plankton contact rates. Journal of Plankton Research, 10, 465–474.

[ece32593-bib-0086] Saiz, E. , & Alcaraz, M. (1992). Free‐swimming behavior of *Acartia clause* (Copepoda: Calanoida) under turbulent movement. Marine Ecology Progress Series, 80, 229–236.

[ece32593-bib-0087] Salehi, M. , & Strom, K. (2011). Using velocimeter signal to noise ratio as a surrogate measure of suspended mud concentration. Continental Shelf Research, 9, 1020–1032.

[ece32593-bib-0088] Samuelsson, P. (2010). Using regional climate models to quantify the impact of climate change on lakes In George G. (Ed.), The impact of climate change on European Lakes (pp. 15–32). Dordrecht: Springer, ISBN 978‐90‐481‐2944‐7.

[ece32593-bib-0089] Scheffer, M. (1998). Ecology of Shallow lakes. London: Chapman & Hall.

[ece32593-bib-0090] Schoener, T. W. (1970). Non‐synchronous spatial overlap of lizards in patchy habitats. Ecology, 51, 408–418.

[ece32593-bib-0091] Schriver, P. , Bøgestrand, J. , Jeppesen, E. , & Søndergaard, M. (1995). Impact of submerged macrophytes on fish‐zooplankton‐phytoplankton interactions: Large‐scale enclosure experiments in a shallow eutrophic lake. Freshwater Biology, 33, 255–270.

[ece32593-bib-0092] Sih, A. , Crowley, P. , McPeek, M. , Petranka, J. , & Strohmeier, K. (1985). Predation, competition, and prey communities. A review of field experiments. Annual Review of Ecology and Systematics, 16, 269–311.

[ece32593-bib-0093] Svanbäck, R. , & Bolnick, D. I. (2007). Interspecific competition drives increased resource use diversity within a natural population. Proceedings of the Royal Society B, 274, 839–844. 17251094 10.1098/rspb.2006.0198 PMC2093969

[ece32593-bib-0094] Tennekes, H. , & Lumley, L. (1972). A first course in turbulence. Cambridge, MA: The MIT Press.

[ece32593-bib-0095] Tilman, D. (1982). Resource competition and community structure. Princeton, NJ, USA: Princeton University Press. 7162524

[ece32593-bib-0096] Urban, M. C. , Tewksbury, J. J. , & Sheldon, K. S. (2012). On a collision course: Competition and dispersal differences create non‐analogue communities and cause extinctions during climate change. Proceedings of the Royal Society B, 279, 2072–2780. 22217718 10.1098/rspb.2011.2367 PMC3311897

[ece32593-bib-0097] Uusitalo, L. , Horppila, J. , Eloranta, P. , Liljendahl‐Nurminen, A. , Malinen, T. , Salonen, M. , & Vinni, M. (2003). *Leptodora kindtii* and flexible foraging behaviour of fish – factors behind the delayed biomass peak of cladocerans in Lake Hiidenvesi. International Review of Hydrobiology, 88, 34–48.

[ece32593-bib-0098] Vašek, M. , Kubečka, J. , Matĕna, J. , & Seda, J. (2006). Distribution and diet of 0+ fish within a canyon‐shaped European reservoir in late summer. International Review of Hydrobiology, 91, 178–194.

[ece32593-bib-0099] Vøllestad, L. A. (1985). Resource partitioning of roach *Rutilus rutilus* and bleak *Alburnus alburnus* in two eutrophic lakes in SE Norway. Holarctic Ecology, 8, 88–92.

[ece32593-bib-0100] Werner, E. E. , & Hall, D. (1974). Optimal foraging and the size selection of prey by the bluegill sunfish (*Lepomis macrochirus*). Ecology, 55, 1042–1052.

[ece32593-bib-0101] Werner, E. E. , & Hall, D. J. (1977). Competition and habitat shift in two sunfishes (Centrachidae). Ecology, 58, 869–876.

[ece32593-bib-0102] Werner, E. E. , & Hall, D. J. (1979). Foraging efficiency and habitat switching in competing sunfishes. Ecology, 60, 256–264.

[ece32593-bib-0103] Windell, J. T. (1971). Food analysis and the rate of digestion In Ricker W. (Ed.), Methods for assessment of fish production in fresh waters IPB handbook 3 (pp. 215–226). Oxford: Blackwell Scientific Publications.

[ece32593-bib-0104] Wrona, F. J. , Prowse, T. D. , Reist, J. D. , Hobbie, H. E. , Lévesque, L. M. J. , & Vincent, W. F. (2006). Climate change effects on aquatic biota, ecosystem structure and function. Ambio, 35, 359–369. 17256640 10.1579/0044-7447(2006)35[359:cceoab]2.0.co;2

